# Clustering of Chromatin Remodeling Enzymes Predicts Prognosis and Clinical Benefit of Therapeutic Strategy in Pancreatic Cancer

**DOI:** 10.7150/ijms.73800

**Published:** 2022-09-21

**Authors:** Hui-Ching Wang, Hsiang-Yao Shih, Chun-Chieh Wu, Li-Tzong Chen, Chi-Wen Luo, Yi-Chang Liu, Jeng-Shiun Du, Min-Chin Huang, Yung-Yeh Su, Huan-Da Chen, Hui-Hua Hsiao, Sin-Hua Moi, Mei-Ren Pan

**Affiliations:** 1 Graduate Institute of Clinical Medicine, College of Medicine, Kaohsiung Medical University, Kaohsiung 807, Taiwan.; 2 Department of Internal Medicine, Division of Hematology and Oncology, Kaohsiung Medical University Hospital, Kaohsiung Medical University, Kaohsiung 807, Taiwan.; 3 Faculty of Medicine, College of Medicine, Kaohsiung Medical University, Kaohsiung 807, Taiwan.; 4 Department of Internal Medicine, Kaohsiung Medical University Hospital, Kaohsiung, Taiwan.; 5 Department of Pathology, Kaohsiung Medical University Hospital, Kaohsiung Medical University, Kaohsiung 807, Taiwan.; 6 National Institute of Cancer Research, National Health Research Institutes, Tainan, Taiwan.; 7 Division of Breast Oncology and Surgery, Department of Surgery, Kaohsiung Medical University Hospital, Kaohsiung 807, Taiwan.; 8 Specialist Nurse and Surgical Nurse Practitioner Office, Kaohsiung Medical University Chung-Ho Memorial Hospital.; 9 Translational Research Center, Kaohsiung Medical University Hospital, Kaohsiung, Taiwan.; 10 Center of Cancer Program Development, E-Da Cancer Hospital, I-Shou University, Kaohsiung 807, Taiwan.; 11 Drug Development and Value Creation Research Center, Kaohsiung Medical University, Kaohsiung, Taiwan.; 12 Department of Medical Research, Kaohsiung Medical University Hospital.

**Keywords:** Epigenetic modification, Pancreatic Cancer, Hierarchical clustering analysis

## Abstract

In recent years, translational research and pharmacological targeting of epigenetic modifications have become the focus of personalized therapy for patients with pancreatic cancer. Preclinical and clinical trials targeting post-translational modifications have been evaluated as monotherapy or in combination with standard chemotherapy. In this study, we selected 43 genes from seven families of chromatin-modifying enzymes and investigated the influences of epigenetic modifications and their interactions on pancreatic ductal adenocarcinoma (PDAC) using hierarchical clustering analysis. Our analysis also evaluated their effects on treatment modalities and regimens of chemotherapy for PDAC. RNA-seq data for a total of 177 patients with pancreatic cancer, obtained from The Cancer Genome Atlas database, were analyzed. Our results suggested that high-risk patients of survival significant chromatin remodeling-associated gene cluster (gene cluster 2), composed of histone methyltransferases, histone acetyltransferases, histone deacetylases, histone demethylases, and 10-11 translocation family, demonstrated inferior progression-free survival and overall survival in patients with PDAC, especially in men. Our novel biomarker, survival significant chromatin remodeling-associated gene cluster, showed superior prediction performance compared with the conventional TNM system. Overall, these findings suggest that epigenetic modifications and interactions play an important role in the prognosis and therapeutic response of patients with PDAC.

## Introduction

Pancreatic ductal adenocarcinoma (PDAC) is one of the malignancies with the worst prognosis worldwide. More than half of the patients have distant metastases at diagnosis, and their five-year survival rate is only 3% 1, 2. PDAC is estimated to become the second leading cause of cancer-related deaths by 2030^3^. Surgery remains the main curative strategy for local or regional PDAC; even after curative surgery, a high distant metastasis rate and local recurrence rate are still observed 4, 5. Different approaches such as adjuvant chemotherapy (CT), radiotherapy (RT), and concurrent chemoradiotherapy have been used to improve survival rates 6, 7. However, there is still no consensus on a standard adjuvant therapy strategy for RT, CT, or a combination of both 8, 9. Adjuvant gemcitabine monotherapy is the preferred regimen in Europe and the United States 10, 11. Adjuvant treatment with combination chemotherapy developed afterward, such as gemcitabine-based chemotherapy and modified FOLFIRINOX, have been recommended in the national guidelines published previously 12-14.

An accumulation of genetic aberrations is well known to cause PDAC malignancy. More than 90% of PDAC cases have a gain-of-function KRAS mutation in the initiating pancreatic intraepithelial neoplasia. During disease progression, several tumor suppressor genes such as TP53, SMAD4, and CDKN2A contribute to and accompany KRAS mutations to promote cancer formation 15, 16. Relative to genetic alternation, epigenetic regulations modify the non-DNA sequence heritably and affect gene expression and genome integrity during the cell cycle 17. Their mechanisms include DNA methylation, histone modification (methylation, acetylation, phosphorylation, ubiquitination, and SUMOylation), chromatin remodeling, and non-coding RNAs. They can be broadly categorized as writers (histone acetyltransferases [HAT], DNA methyltransferases [DNMT], and histone methyltransferases [HMT]), readers (bromodomains, tudor domains, PHD fingers, and chromodomains), and erasers (histone deacetylases [HDAC], histone demethylases [HDM], and 10-11 translocation [TET] family of dioxygenases), which not only apply to molecules that modify histones but also to DNA methylation 18, 19. In recent years, the important roles of epigenetic alterations and modulations have begun to emerge in different types of cancer 20, 21.

Increasingly, epigenetic aberrations are being implicated in malignancies of pancreatic cancer. For example, previous studies found that the transcription factor FOXA1 in PDAC cells could activate the GAIN enhancer region via acetylation of H3K27 (lysine 27 of histone 3) and facilitate anoikis-resistant cell growth 22, 23. EZH2, a catalytic subunit of polycomb repressive complex 2 (PRC2), serves as an HMT responsible for the methylation of H3K27 and suppresses target gene expression, ultimately leading to carcinogenesis of intraductal papillary mucinous neoplasms of the pancreas 24, 25. G9a (or EHMT2), another famous HMT, methylates the 9 and 27 lysine residues of histone H3 (H3K9 and H3K27), represses the transcription of tumor suppressor genes, and promotes tumor invasion and metastasis 26, 27. In addition, different functions of chromatin-modifying enzymes also affect different regulations; for example, mutations in genes that encode KDM6A, an eraser of the H3K27me3 mark, and MLL2, a writer of H3K4me3, were elucidated in whole-genome sequencing of human pancreatic cancers 16. To tackle this problem, drugs targeting epigenetic aberrations in pancreatic cancer have been developed in recent decades 28, 29. In summary, the epigenomic landscapes in PDAC may provide different perspectives for reassessing this disease.

In this study, we aimed to evaluate the association between epigenetic modifications and clinical outcomes as well as treatment strategies for PDAC. We used a hierarchical clustering method to assess the influence of baseline characteristics, genomic profiles, and treatment modalities on the OS and outcomes of patients with PDAC.

## Methods

### Data source

All data were downloaded from TCGA Pan-Cancer Pancreatic ductal adenocarcinoma (PAAD) project via the cBioPortal website (https://www.cbioportal.org/study/summary) 30, 31. This study included 177 patients with pancreatic cancer for whom RNA-seq expression data were available. Baseline characteristics included age, ethnicity, race, histology, pathological stage, lymph node invasion, pathological stage, and treatment characteristics. The treatment characteristics of the study population were categorized into multiple subgroups according to their RT and CT status, and gemcitabine-based CT was considered an independent subgroup in addition to other CT. Therefore, the treatment characteristics of the study population were finally categorized into multiple subgroups: RT alone, CT (Gemcitabine-based) alone, CT (Others) alone, RT combined with CT (Gemcitabine-based), RT combined with CT (Others), and the remaining were considered as none of the RT and CT subgroups. Progression-free survival (PFS) and overall survival (OS) were considered survival outcomes for the study population. All patients were tracked from the date of initial diagnosis until the date of disease progression, metastases, or the end of the study.

### Candidate gene profiling

#### Gene expression level

We analyzed 43 genes from seven families of chromatin modification enzymes composed of HMT family including SUV39H1, SUV39H2, EHMT2 (euchromatic histone lysine methyltransferase 2), EHMT1 (euchromatic histone lysine methyltransferase 1), SETDB1 (SET domain bifurcated histone lysine methyltransferase 1), SETDB2 (SET domain bifurcated histone lysine methyltransferase 2), EZH2 (enhancer of zeste 2 polycomb repressive complex 2 subunit), EZH1 (enhancer of zeste 2 polycomb repressive complex 2 subunit), KMT 5A (lysine methyltransferase 5A, also known as SETD8), KMT5B (lysine methyltransferase 5B, also known as SUV420H1), and KMT5C (lysine methyltransferase 5C, also known as SUV420H2); HAT family including EP300 (E1A binding protein p300), CREBBP (CREB binding protein, also known as CBP), KAT2A (lysine acetyltransferase 2A), and KAT7 (lysine acetyltransferase 7, also known as MYST2), KAT8 (lysine acetyltransferase 8, also known as MYST1); BET family including BRD2 (bromodomain containing 2), BRD3 (bromodomain containing 3), BRD4 (bromodomain containing 3), and BRDT (bromodomain testis associated); HDAC family including HDAC1 (histone deacetylase 1), HDAC2 (histone deacetylase 2), HDAC3 (histone deacetylase 3), HDAC4 (histone deacetylase 4), HDAC6 (histone deacetylase 6), HDAC7 (histone deacetylase 7), SIRT1 (sirtuin 1), and SIRT2 (2); HDM family including KDM1A (lysine demethylase 1A), KDM3A (lysine demethylase 3A), KDM3B (lysine demethylase 3B), KDM4A (lysine demethylase 4A), KDM4B (lysine demethylase 4B), KDM5C (lysine demethylase 5C), KDM5D (lysine demethylase 5D), KDM6B (lysine demethylase 6B), and KDM8 (lysine demethylase 8, also known JMJD5); DNMT family including DNMT1 (DNA methyltransferase 1), DNMT3A (DNA methyltransferase 3A), and DNMT3B (DNA methyltransferase 3B); TET family including TET1 (tet methylcytosine dioxygenase 1), TET2 (tet methylcytosine dioxygenase 2), and TET3 (tet methylcytosine dioxygenase 3).

The gene expression levels of candidate genes were estimated using log-transformed mRNA expression z-scores compared to the expression distribution of all samples (RNA Seq V2 RSEM). The original RNA-seq expression of each candidate gene is illustrated using a boxplot according to the PFS and OS status, and the difference in mRNA expression between subgroups was tested using the Wilcoxon rank sum test.

#### Gene clustering and risk subgroups identification

Candidate genes were clustered into multiple clusters based on their similarity using a hierarchical clustering algorithm. First, the RNA-seq expression of the candidate genes was normalized to a range between 0 and 1. The average silhouette width was calculated to obtain the optimal cluster number for gene clustering; a greater width indicated greater dissimilarity between the determined clusters. All candidate genes were then agglomerated according to their similarity and visualized using a dendrogram. Next, candidate genes were clustered into *k* clusters according to the agglomerative order shown in the dendrogram. The normalized RNA-seq expression of each gene involved in *k*th clusters was then used in the risk subgroups identification procedure. A distance matrix between each sample was generated using the normalized RNA-seq expression of each gene in the *k*th cluster. The samples with the closest distance will be merged until all samples were dichotomized into two risk subgroups. Afterward, the risk subgroups were defined as low-and high-risk subgroups according to the proportion of PFS and OS between the two subgroups.

### Statistical analysis

The RNA-seq expression of each gene is summarized as the median and interquartile range (IQR) according to the risk subgroup determined by the corresponding gene cluster. The difference in gene expression among the risk subgroups was estimated using the Wilcoxon rank-sum test. The heatmap of the determining gene cluster was visualized and annotated with the risk subgroup, sex, PFS, and OS. The correlation between candidate genes was visualized using a scatter plot, and the correlation coefficient was computed using the Pearson correlation test. The baseline characteristics of the study population according to the risk subgroup determined by the corresponding gene cluster were presented as frequency and percentage, and the diagnosis age was summarized as median and IQR. The differences in the baseline characteristics were estimated using the Wilcoxon rank-sum test, chi-squared test, and Fisher's exact test. The survival outcomes of the study population were illustrated using the Kaplan-Meier method, and the survival difference between subgroups was estimated using the log-rank test. Furthermore, the association between survival outcomes, and each clinical characteristic, or gene cluster risk subgroup was estimated using univariate Cox regression. A multivariate Cox regression model was generated using stepwise selection, the final multivariate model could interpret the impacted factors associated with survival outcomes. All *p values* were two-sided, and statistical significance was set at* p < 0.05*. All analyses were performed using the R 4.0.5 software (R Core Team, 2021).

## Results

### Clinicopathological Characteristics and Progression of Pancreatic Cancer

Seven families of chromatin-modifying enzymes are listed in Table 1. The clinicopathological characteristics of 177 patients with pancreatic cancer were collected and summarized from TCGA-PAAD of the GDC data portal in Table 2. The RNA-seq expression of each gene according to PFS and OS status is illustrated using boxplots in Supplementary Figures S1 and S2, respectively. In the HMT family, *EZH1*, *EZH2*, and *SETDB2* showed significantly different expressions in both PFS and OS, while *EHMT2* only showed significantly different expressions in PFS. In the HAT family, *KAT2A* and *MYST1* were significantly differentially expressed in both PFS and OS. In the HDAC family, *HDAC6* showed significantly different expression in both PFS and OS status, while *SIRT2* showed only significantly different expression in PFS status, and *HDAC3* and *HDAC4* showed only significantly different expression in OS status. In the HDM family, *JMJD5*, *KDM6B*, and *KDM4B* showed significantly different expressions in both PFS and OS, while *KDM1A* and *KDM5D* showed significantly different expressions in OS. The* TET3* gene from the TET family showed only significantly different expression in PFS status. Moreover, the RNA-seq expression of all genes from the BET and DNMT families showed no significant differences in either PFS or OS status.

### Agglomerative Hierarchical Clustering Analysis

**Figure 1:**
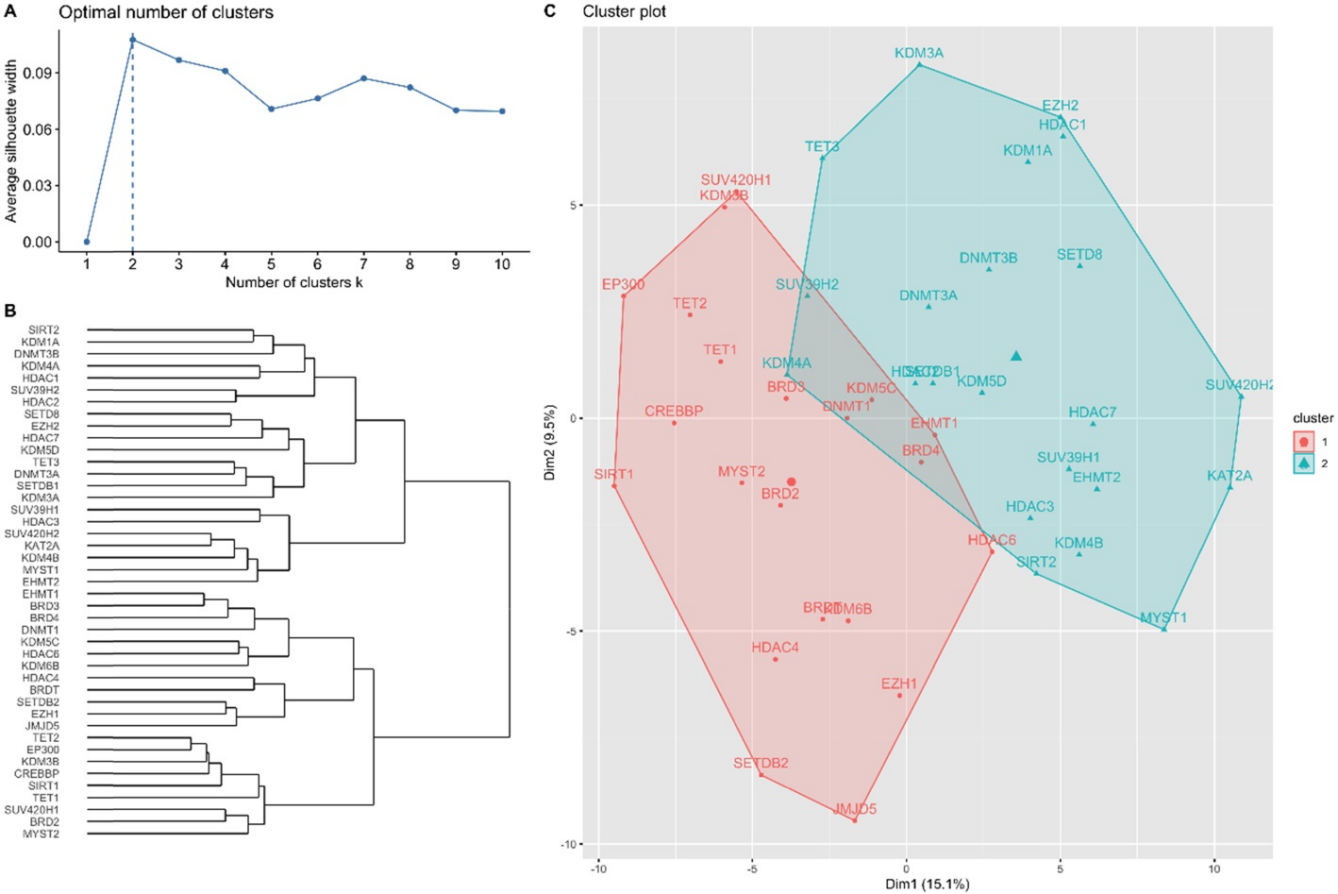
Hierarchical clustering analysis results for candidate genes. (A) Optimal number of clusters determined using average silhouette width. (B) Dendrogram of agglomerative hierarchical clustering according to the similarity between candidate genes. (C) Cluster plot of optimal gene cluster determined by the hierarchical clustering algorithm.

The results of the hierarchical clustering analysis are summarized in Figure 1. The optimal number of clusters was two, determined using the average silhouette width (Figure 1A). Figure 1B shows a dendrogram of the candidate genes by the agglomerative clustering results. Accordingly, *BRD2*, *BRD3*, *BRD4*, *BRDT*, *CREBBP*, *DNMT1*, *EHMT1*, *EP300*, *EZH1*, *HDAC4*, *HDAC6*, *JMJD5*, *KDM3B*, *KDM5C*, *KDM6B*, *MYST2*, *SETDB2*, *SIRT1*, *SUV420H1*, *TET1*, and *TET2* were clustered in gene cluster 1 (GC1). *DNMT3A*, *DNMT3B*, *EHMT2*, *EZH2*, *HDAC1*, *HDAC2*, *HDAC3*, *HDAC7*, *KAT2A*, *KDM1A*, *KDM3A*, *KDM4A*, *KDM4B*, *KDM5D*, *MYST1*, *SETD8*, *SETDB1*, SIRT2, *SUV39H1*, *SUV39H2*, *SUV420H2*, and *TET3* were clustered in gene cluster 2 (GC2). The RNA-seq expression of each gene in both gene clusters is summarized in Tables 3 and 4. The results showed that most genes showed significantly different expression in the low- and high-risk subgroups in GC1, except for *BRDT*, *HDAC4*, *JMJD5*, and *SETDB2*. Similarly, most genes in GC2 showed significantly different expression levels in the low- and high-risk subgroups, except for* DNMT3A*, *DNMT3B*,* EZH2*, *HDAC1*,* HDAC2*,* KDM1A*, *KDM3A*,* KDM5D*, and* SETDB1*. The baseline characteristics of the study population according to each gene cluster are summarized in Tables 5 and 6. No significant differences in the distribution of baseline characteristics were found in the risk subgroup of GC1. However, the high-risk subgroup of GC2 showed a significantly higher proportion of progressive disease (high-risk vs. low-risk, 78.8% vs. 53.5%, *p* = 0.008) and death (high-risk vs. low-risk, 81.8% vs. 45.1%, *p* < 0.001) events.

**Figure 2:**
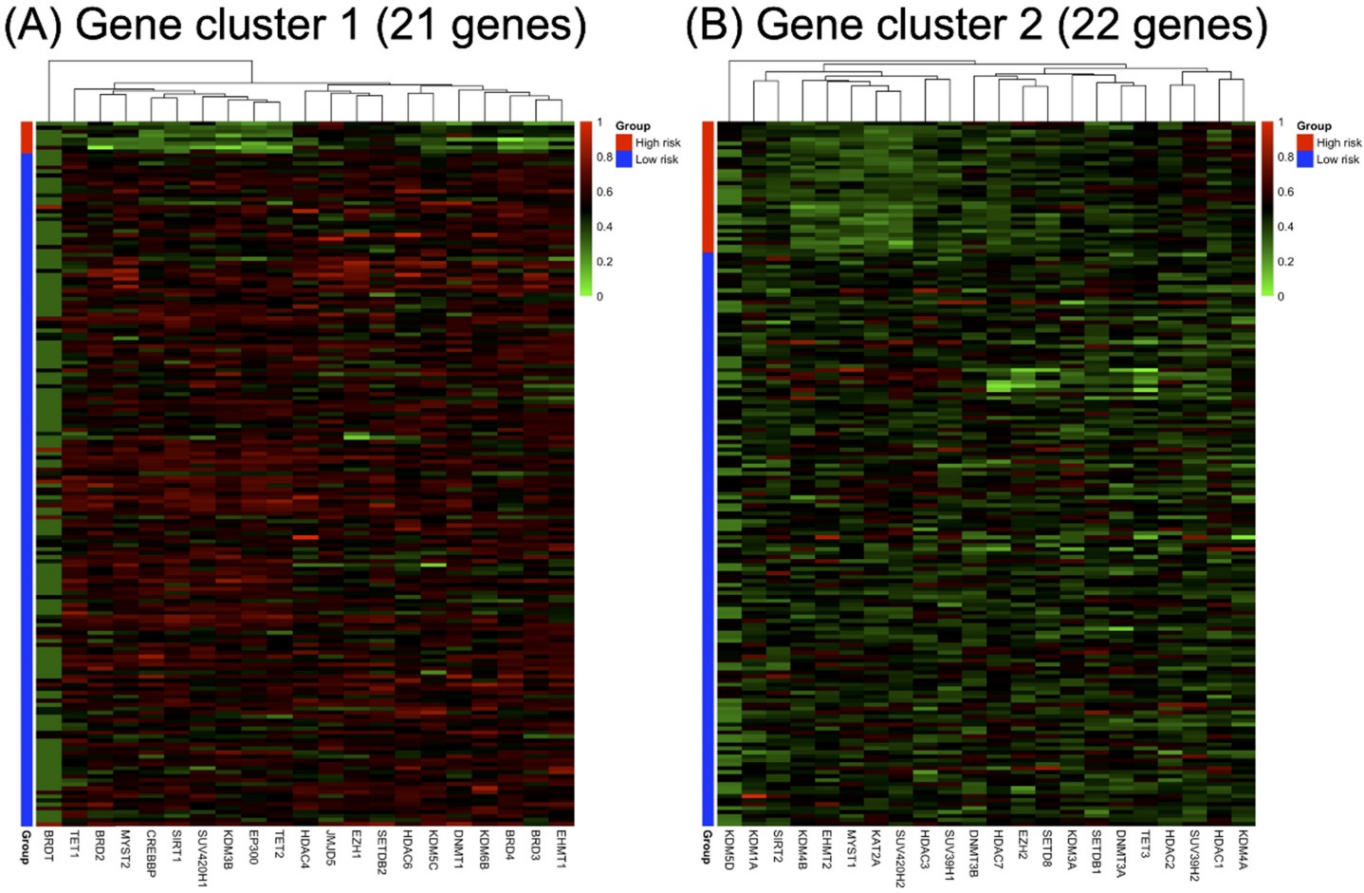
Annotated heatmap of candidate genes of (A) gene cluster 1 and (B) gene cluster 2 based on RNA-seq expression. All heatmaps were ordered according to the risk subgroup, sex, progression-free survival (PFS), and overall survival (OS) status. The red color indicates a higher expression level, green color indicates a lower expression level of RNA-seq.

**Figure 3:**
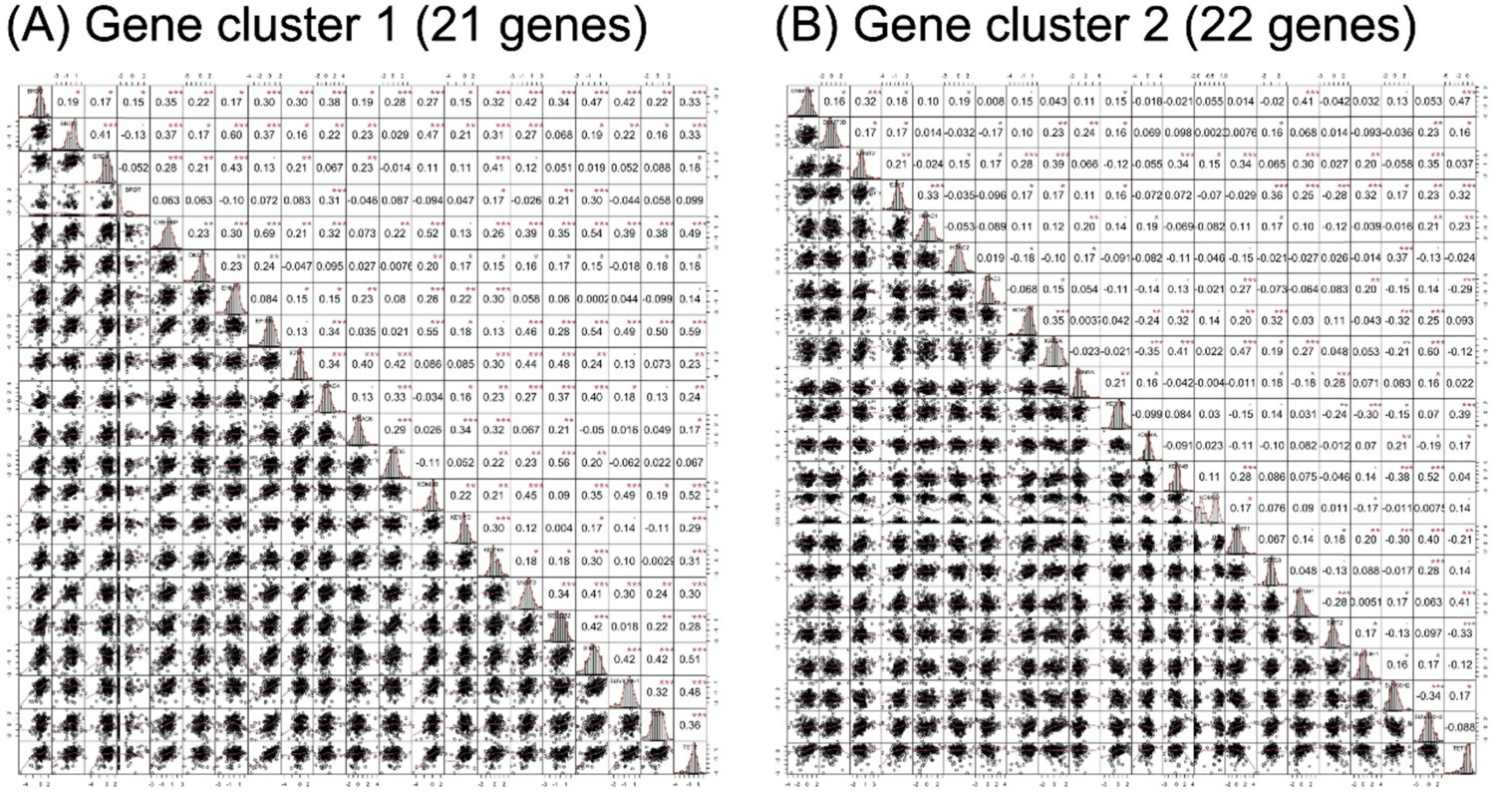
Correlation analysis results of candidate genes in (A) gene cluster 1 and (B) gene cluster 2. The lower triangular area shows the scatter plot of pairwise genes, and the upper triangular area shows the correlation coefficients of each pair. The middle diagonal cell revealed the histogram of each gene according to the distribution of RNA-seq expression in the study population. ^•^*p* < 0.1, *p < 0.05, **p < 0.01, ***p < 0.001.

RNA-seq expression of the included genes in each gene cluster is illustrated in Figure 2. The heat maps were annotated and ordered by risk subgroup, sex, PFS, and OS status. Red indicates a higher expression level, while green indicates a lower expression level in the RNA-seq. As shown in Figure 2A, the high-risk subgroup of GC1 showed lower expression of *BRD2*, *BRD3*, *BRD4*, *CREBBP*, *DNMT1*, *EHMT1*, *EP300*, *HDAC6*, *KDM3B*, *KDM5C*, *KDM6B*, *MYST2*, *SIRT1*, *SUV420H1*, *TET1*, and *TET2*. We also found lower RNA expression for *KDM4B*, *EHMT2*, *MYST1*, *KAT2A*, *HDAC3*, *SUV39H1*, and *SUV420H2* in the high-risk subgroup of GC2 (Figure 2B). The pairwise correlation between each candidate gene is summarized in Figure 3. Most of the genes were significantly correlated with genes within or across different gene families, which indicates a potential co-regulated role between candidate genes included in the current study.

### Clustering-based Risk Subgroups: Impact on Survival Outcomes

**Figure 4:**
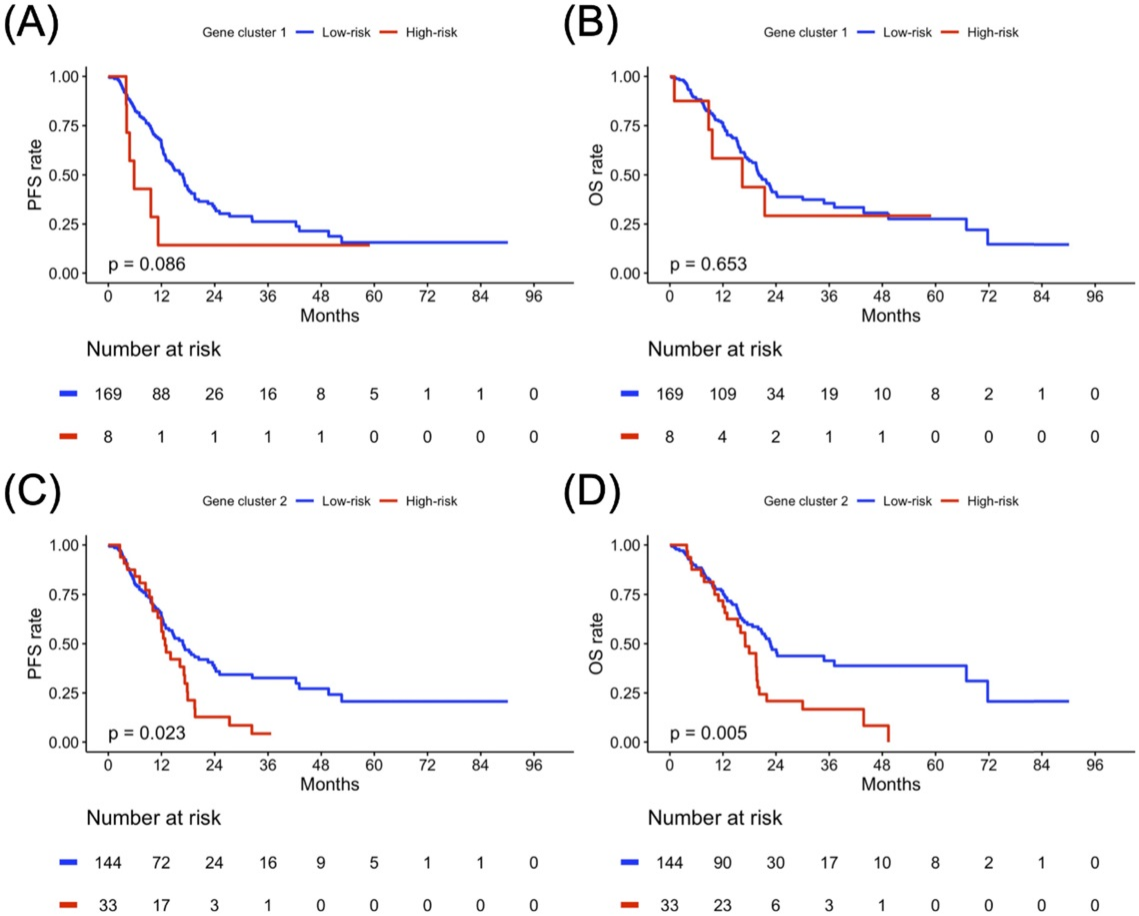
Progression-free survival (PFS) and overall survival (OS) analyses result in gene clusters of the study population. Kaplan-Meier plot for (A) PFS and (B) OS of gene cluster 1. Kaplan-Meier plot for (C) PFS and (D) OS of gene cluster 2. The red solid line indicates the high-risk subgroup, and the blue solid line indicates the low-risk subgroup. *P*-value is estimated using the log-rank test.

**Figure 5:**
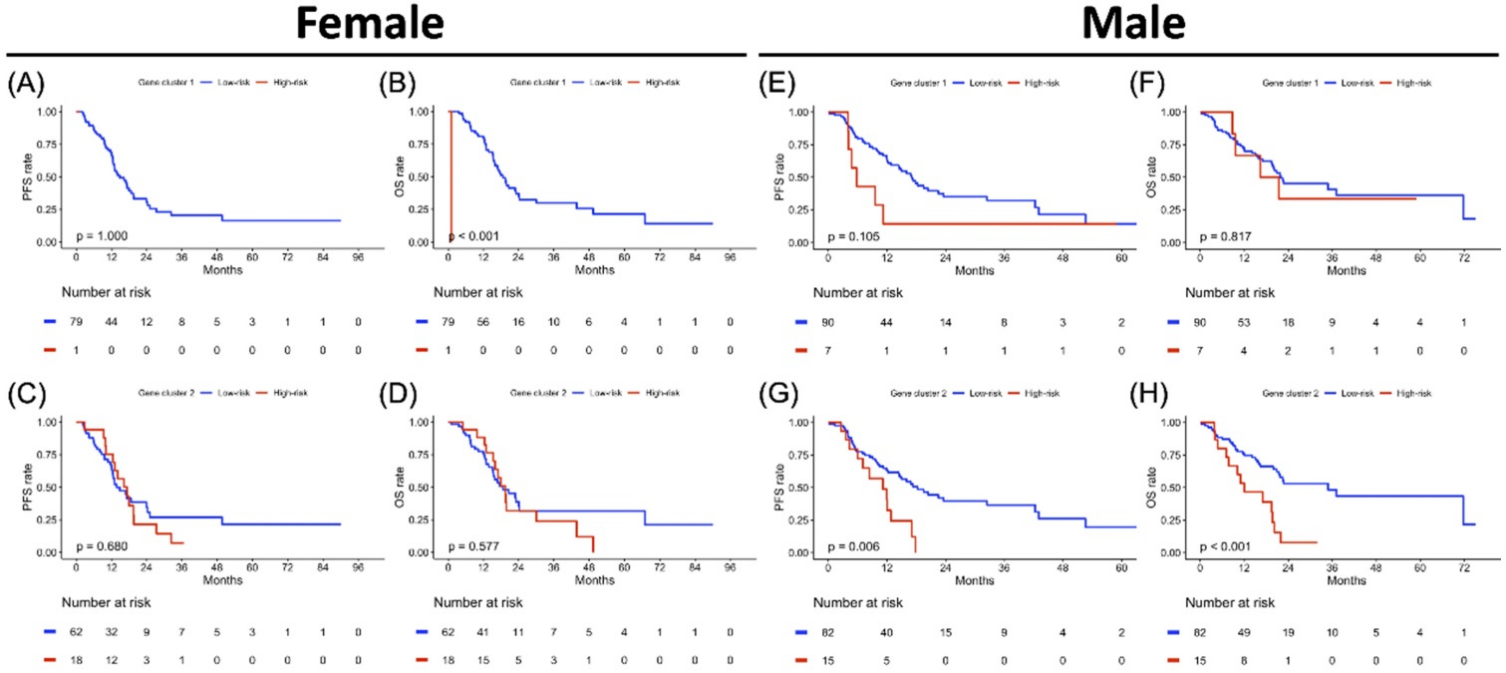
Sex-specified progression-free survival (PFS) and overall survival (OS) analyses results in two gene clusters of the study population. Kaplan-Meier plot for (A) PFS and (B) OS of gene cluster 1, (C) PFS and (D) OS of gene cluster 2 in the female cohort. Kaplan-Meier plot for (E) PFS and (F) OS of gene cluster 1, (G) PFS and (H) OS of gene cluster 2 in a male cohort. The red solid line indicates the high-risk subgroup, and the blue solid line indicates the low-risk subgroup. *P*-value is estimated using the log-rank test.

**Figure 6:**
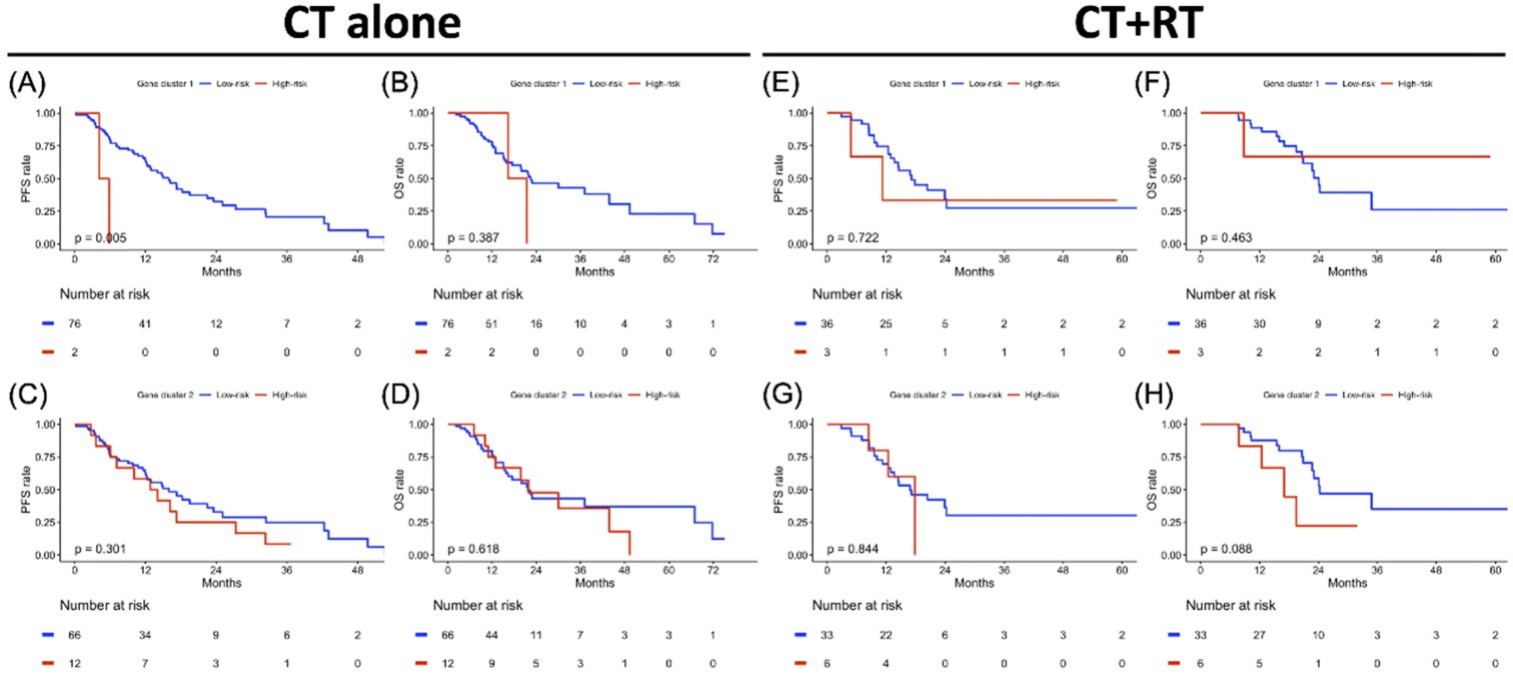
Treatment-specified progression-free survival (PFS) and overall survival (OS) analyses result in gene clusters of radiotherapy and chemotherapy cohorts each. Kaplan-Meier plot for (A) PFS and (B) OS of gene cluster 1, (C) PFS and (D) OS of gene cluster 2 in the CT cohort alone. Kaplan-Meier plot for (E) PFS and (F) OS of gene cluster 1, (G) PFS and (H) OS of gene cluster 2 in CT+RT cohort. The red solid line indicates the high-risk subgroup, and the blue solid line indicates the low-risk subgroup. *P*-value is estimated using the log-rank test.

The PFS and OS analysis results for the risk subgroup in each gene cluster of the study population are illustrated in Figure 4. The high-risk subgroup in both GC1 and GC2 showed poor survival outcomes compared to the low-risk subgroup, but only the high-risk subgroup of GC2 showed significantly worse survival outcomes compared to the low-risk subgroup of GC2 (Figure 4C, *p* = 0.023; Figure 4D, *p* = 0.005). To further clarify the impact of the determined risk subgroup on sex-specific survival outcomes, Figure 5 presents the survival analysis results for risk subgroups of each gene cluster in the female and male cohorts. Similarly, the high-risk subgroup still had poor survival outcomes compared to the low-risk subgroup in both GC1 and GC2. Notably, the high-risk subgroup of GC2 showed a significantly worse survival outcome than that of the low-risk subgroup (Figure 5G, *p* = 0.006; Figure 5H, *p* < 0.001). As shown in Figure 6, although the high-risk subgroup still had poor survival outcomes compared to the low-risk subgroup in both GC1 and GC2, no significant survival differences were observed in different treatment cohorts. However, compared to the high-risk subgroup derived using GC2, the low-risk subgroup demonstrated better PFS and OS, especially in patients treated with RT combined with CT. Table 7 interpreted the Cox regression analysis results for PFS and OS. GC2 remained a significant impact on PFS, but an insignificant impact on OS. The OS outcomes were significantly associated with age, histology, LN, and treatment subgroup rather than gene profiles. It is worth noting that we used the forest plot for risk estimation based on gene expression associated with chromatin remodeling, showing the effect size and significance of each observation associated with chromatin remodeling. We separate the estimated result into two parts according to the measured outcomes, including the disease progression (PFS or DFS) and OS. All measurements were abstracted from the multivariate Cox regression model based on the cancer population. In disease progression outcomes, both gene clusters derived from the current study were included. The comparison results show GC2 could obtain greater weight in both common-effect and random-effects models, compared with GC1 or TET families 32, except for TET3 expression in acute myeloid leukemia (AML) study 33. Since our multivariate Cox regression model for OS included only GC2, so we only compared the genes involved in GC2. A similar finding was also found in OS outcome, GC2 obtained greater weight in both common-effect and random-effects model, compared with TET3 in breast cancer study 34, but lower than TET3 expression in AML study 33. In summary, the forest plot comparison results showed that GC2 might obtained potential risk estimation effects for the study population in both disease progression and OS outcomes, compared to the single gene expression model (Supplementary Figure S3).

## Discussions

The major contribution of this study is the use of a hierarchical clustering approach to analyze the epigenetic profile of PDAC. First, we selected 43 candidate genes that belonged to seven types of chromatin-modifying enzymes and divided them with the hierarchical clustering method into two subgroups. We also categorized the 43 candidate genes according to PFS and OS status using boxplots. Second, we identified low- and high-risk patients based on mRNA expression levels derived from two epigenetic gene clusters according to PFS and OS status. The study results revealed that GC2 is considered a survival significant chromatin remodeling-associated gene cluster. In the identified survival significant chromatin remodeling-associated gene cluster, there was no significant clinicopathological characteristics difference between the high-and low-risk groups, except for race, disease progression, and death events. Third, we confirmed the potential interaction and regulation between different gene families using Pearson's correlation test. Fourth, we identified the predictive role of survival significant chromatin remodeling-associated gene cluster for PFS and OS using Kaplan-Meier analysis, especially in men. Finally, we evaluated the therapeutic response to different treatment strategies using survival significant chromatin remodeling-associated gene cluster. Although the predictive efficacy of the response was not obvious, the study results still demonstrated a better response to RT combined with CT in significant chromatin remodeling-associated gene cluster survival.

Consistent with previous reports on different cancer types, we found a correlation between mRNA levels of chromatin-modifying enzymes and clinical outcomes. For example, the reduction of MYST1, also known as hMOF protein, in renal cell carcinoma is correlated with the acetylation of histone H4K16, implicating MYST1 in the pathogenesis of kidney cancer 35. Reduced SIRT2 expression promotes serious ovarian carcinoma migration and invasion, suggesting that SIRT2 may serve as a tumor suppressor and a therapeutic target in ovarian cancer 36. In cervical cancer cells, cells with low levels of SUV39H1 protein have a higher migratory ability *in vitro*, and SUV39H1 knockdown *in vitro* enhances cancer cell migration 37. Loss of SUV420H2 facilitates upregulation of LINC01510, which promotes the transcription of the oncogene MET and EGFR inhibitor resistance in lung cancer 38. KDM4A serves as a poor prognostic marker and plays an oncogenic role in oral squamous cell carcinoma and nasopharyngeal cancer 39, 40. In contrast to SUV39H1, SUV39H2 expression is elevated and might be a potential oncogene that mediates tumorigenesis and metastasis in lung adenocarcinoma 41. KDM4B promotes EMT through the upregulation of ZEB1 in PDAC cells 42. However, these studies analyzed genes or proteins from one of the chromatin-modifying enzymes individually.

Chromatin-modifying enzymes communicate and interact with each other 43, 44. Cross-talk between different histone modifications also controls chromatin structure 45. For example, HAT leads to chromatin relaxation and may facilitate the accessibility of DNA to transcription factors and the whole transcriptional machinery; conversely, HDACs are associated with gene repression. Both groups dynamically interact and regulate gene expression through physical interactions with sequence-specific transcription factors 46. SET1C-mediated H3K4me3 is enhanced by p53- and p300-dependent H3 acetylation, which indicates a connection between HAT and HMT mediated by the p300 bromodomain 47. Moreover, crosstalk is involved in the interaction between genomic, epigenomic, and signaling pathway alterations 48. Loss of AIRID1A, a member of the SWI/SNF complex, regulates downstream PI3K-AKT signaling via the PI3K-interacting protein 1 gene (PIK3IP1). Using an EZH2 inhibitor reinforces the synthetic lethal strategy by upregulating PIK3IP1 in AIRID1A-mutated cancers 49. Additionally, the tumor microenvironment could be modulated by chromatin remodeling 50. Epigenetic regulation affects cytokine secretion and immune cell recruitment, shaping the tumor microenvironment and influencing the outcome of immunotherapy. Therefore, an overall assessment of chromatin-modifying enzymes and their interactions has recently become crucial. To the best of our knowledge, no existing analysis has assessed a group of chromatin-modifying enzymes as predictors of clinical outcomes. The results of our analysis not only showed consistent a trend of their biological functions but also conveyed a message for clinical significance that our analysis may be a potential way to stratify different risks of pancreatic cancer patients.

In this study, we used the hierarchical clustering approach to identify the similarity between genes from different families of epigenetic regulators, and integrate the survival association between clustered genes. The involved genes were divided into two clusters which indicate the expression between the clustered genes has a large dissimilarity in the study population. Nevertheless, our study has some limitations. The dataset derived from the TCGA database recruits most patients from Western countries. Most patients with PDAC are in a relatively early stage, especially stage II. In addition, the small sample size might restrict the expression of findings in the study population, and the random effects might also be a potential bias. Fig S1 and Fig S2 showed that survival significant effects might not be present in most of the single gene expressions, and hence, the hierarchical clustering depending on the similarity of multiple genes expression might contribute to integrating the co-expression associated with survival outcomes. In both identified gene clusters, the numbers of patients at high and low risk were not equally distributed, which may have led to statistical bias. The distribution of patient numbers was not even in different chemotherapy (gemcitabine-or non-gemcitabine-based) or therapeutic strategies (CT alone or CT combined with RT). More *in vivo* and *in vitro* studies are required to confirm these computational results.

## Conclusions

This is the first study to analyze the impact of epigenetic modifications and their interactions with clinical outcomes and treatment modalities using a hierarchical clustering algorithm in patients with PDAC. The study findings suggest that high-risk patients of survival significant chromatin remodeling-associated gene cluster, composed of the HMT, HAT, HDAC, HDM, and TET families, demonstrated inferior PFS and OS in patients with PDAC, especially in men. In addition, our results suggest that these patients may benefit from chemotherapy combined with radiotherapy, rather than chemotherapy alone. Our novel biomarker, survival significant chromatin remodeling-associated gene cluster, showed superior prediction performance compared with the conventional TNM system. Therefore, the overall findings suggest that epigenetic modifications and interactions play a pivotal role in the prognosis and therapeutic response. However, a more complete pathophysiological approach is warranted to illustrate the complex relationship between prognostic epigenetic alterations and treatment modalities to encourage precise prediction of PDAC.

## Supplementary Material

Supplementary figures.

## Figures and Tables

**Table 1:** Seven families of chromatin modification enzymes.

Family	Members
HAT	EP300, CBP, KAT2A, KAT7, KAT8
DNMT	DNMT1, DNMT3A, DNMT3B
HMT	SUV39H1 (H3K9 HMT), SUV39H2 (H3K9 HMT), EHMT1 (H3K9 HMT), EHMT2 (H3K9 HMT), SETDB1 (H4K20 HMT), SETDB2 (H4K20 HMT), EZH2 (H3K27 HMT), EZH1 (H3K27 HMT), SETD8 (H3K4 HMT), SUV420H1 (H4K20 HMT), SUV420H2 (H4K20 HMT)
HDAC	HDAC1, HDAC2, HDAC3, HDAC4, HDAC6, HDAC7, SIRT1, SITR2
HDM	KDM1A (H3K4 and H3K9 HDM), KDM3A (H3K9 HDM), KDM3B (H3K9 HDM), KDM4A (H3K9 HDM), KDM4B (H3K9 HDM), KDM5C (H3K4 HDM), KDM5D (H3K4 HDM), KDM6B (H3K27 HDM), KDM8 (H3K36 HDM)
TET	TET1, TET2, TET3
BET	BRD2, BRD3, BRD4, BRDT

**Table 2:** Baseline characteristics of study population (n = 177).

Characteristics	Overall, n=177
Diagnosis age	65 (35 - 88)
Sex	
Female	80 (45.2%)
Male	97 (54.8%)
Ethnicity	
Hispanic Or Latino	5 (2.8%)
Not Hispanic Or Latino	130 (73.4%)
Unknown	42 (23.7%)
Race	
Asian	11 (6.2%)
Black or African American	6 (3.4%)
White	156 (88.1%)
Unknown	4 (2.3%)
Histology	
8500/3	142 (80.2%)
Others	35 (19.8%)
Pathological stage	
Stage 1	21 (12.0%)
Stage 2	146 (83.4%)
Stage 3	4 (2.3%)
Stage 4	4 (2.3%)
Unknown	2
LN+	123 (69.5%)
Treatment	
CT (Gemcitabine-based)	70 (39.5%)
RT+CT (Gemcitabine-based)	24 (13.6%)
CT (Others)	8 (4.5%)
RT+CT (Others)	15 (8.5%)
RT alone	4 (2.3%)
None	56 (31.6%)
Progressed disease	103 (58.2%)
Died	92 (52.0%)

**Table 3:** RNA-seq expression of candidate genes involved in gene cluster 1 (n=177).

Characteristics	Overall, n=177	Gene cluster 1 (GC1)		
		Low-risk, n = 169	High-risk, n = 8	*P*
GC1 included genes				
BRD2	0.02 (-5.42 - 2.60)	0.07 (-2.25 - 2.60)	-1.02 (-5.42 - -0.28)	**<0.001**
BRD3	0.26 (-3.65 - 2.42)	0.31 (-2.45 - 2.42)	-2.35 (-3.65 - -0.94)	**<0.001**
BRD4	0.11 (-5.28 - 2.24)	0.14 (-2.41 - 2.24)	-1.90 (-5.28 - -0.52)	**<0.001**
BRDT	-2.37 (-2.37 - 2.78)	-2.37 (-2.37 - 2.78)	-2.37 (-2.37 - 0.54)	0.858
CREBBP	0.13 (-3.07 - 2.83)	0.20 (-2.27 - 2.83)	-2.68 (-3.07 - -1.26)	**<0.001**
DNMT1	0.07 (-3.62 - 2.95)	0.08 (-2.42 - 2.95)	-1.28 (-3.62 - 1.53)	**0.003**
EHMT1	0.14 (-3.33 - 2.40)	0.17 (-3.20 - 2.40)	-1.06 (-3.33 - -0.49)	**<0.001**
EP300	0.10 (-3.88 - 1.87)	0.18 (-2.77 - 1.87)	-2.84 (-3.88 - -0.96)	**<0.001**
EZH1	-0.05 (-4.53 - 3.07)	0.03 (-4.53 - 3.07)	-0.80 (-1.29 - 0.23)	**0.014**
HDAC4	-0.01 (-2.67 - 4.45)	0.01 (-2.67 - 4.45)	-0.65 (-2.40 - 0.79)	0.060
HDAC6	0.00 (-2.19 - 4.07)	0.03 (-2.14 - 4.07)	-0.98 (-2.19 - -0.47)	**<0.001**
JMJD5	0.03 (-2.64 - 3.60)	0.06 (-2.64 - 3.60)	-0.43 (-2.38 - 1.44)	0.514
KDM3B	0.18 (-4.75 - 2.59)	0.24 (-2.13 - 2.59)	-2.13 (-4.75 - -1.70)	**<0.001**
KDM5C	-0.08 (-4.60 - 2.74)	-0.05 (-4.60 - 2.74)	-1.78 (-2.52 - -0.98)	**<0.001**
KDM6B	-0.12 (-3.52 - 3.05)	-0.10 (-2.16 - 3.05)	-0.89 (-3.52 - -0.52)	**<0.001**
MYST2	0.03 (-3.19 - 2.99)	0.07 (-2.88 - 2.99)	-1.83 (-3.19 - -0.15)	**<0.001**
SETDB2	0.03 (-3.09 - 2.60)	0.08 (-3.09 - 2.60)	-0.71 (-0.91 - 0.42)	0.128
SIRT1	0.05 (-2.98 - 2.13)	0.10 (-2.41 - 2.13)	-2.28 (-2.98 - -1.14)	**<0.001**
SUV420H1	0.07 (-3.76 - 2.03)	0.07 (-2.90 - 2.03)	-2.08 (-3.76 - 0.57)	**<0.001**
TET1	0.07 (-2.78 - 2.62)	0.14 (-2.55 - 2.62)	-1.23 (-2.78 - 0.24)	**0.003**
TET2	0.16 (-4.09 - 2.52)	0.17 (-3.00 - 2.52)	-2.53 (-4.09 - -0.78)	**<0.001**

*P*-value is estimated using Wilcoxon rank-sum test.

**Table 4:** RNA-seq expression of candidate genes involved in gene cluster 2 (n=177).

Characteristics	Overall, n=177	Gene cluster 2 (GC2)		
		Low-risk, n = 144	High-risk, n = 33	*P*
GC2 included genes				
DNMT3A	0.17 (-3.83 - 2.14)	0.20 (-3.83 - 2.14)	-0.07 (-1.98 - 1.45)	0.154
DNMT3B	-0.03 (-2.40 - 3.68)	-0.03 (-2.40 - 3.68)	-0.06 (-2.16 - 1.75)	0.292
EHMT2	0.02 (-2.39 - 4.51)	0.22 (-1.67 - 4.51)	-1.04 (-2.39 - 0.65)	**<0.001**
EZH2	-0.04 (-3.92 - 3.07)	-0.01 (-3.92 - 3.07)	-0.11 (-1.77 - 2.16)	0.235
HDAC1	-0.11 (-2.39 - 2.98)	-0.11 (-2.39 - 2.98)	-0.12 (-1.71 - 1.84)	0.568
HDAC2	0.01 (-2.87 - 3.33)	-0.06 (-2.87 - 3.33)	0.04 (-1.21 - 1.30)	0.408
HDAC3	-0.05 (-2.76 - 4.11)	0.07 (-2.76 - 4.11)	-0.47 (-1.38 - 0.45)	**<0.001**
HDAC7	0.18 (-4.39 - 1.92)	0.39 (-4.39 - 1.92)	-0.70 (-1.96 - 0.23)	**<0.001**
KAT2A	-0.03 (-2.52 - 2.31)	0.25 (-1.56 - 2.31)	-1.22 (-2.52 - 0.14)	**<0.001**
KDM1A	-0.11 (-2.42 - 5.90)	-0.13 (-2.42 - 5.90)	-0.03 (-1.58 - 1.57)	0.823
KDM3A	0.01 (-3.61 - 2.71)	0.01 (-3.61 - 2.67)	0.11 (-1.01 - 2.71)	0.426
KDM4A	0.06 (-4.52 - 4.23)	-0.05 (-4.52 - 4.23)	0.45 (-1.09 - 2.32)	**<0.001**
KDM4B	0.03 (-3.08 - 4.01)	0.22 (-2.73 - 4.01)	-0.95 (-3.08 - 1.12)	**<0.001**
KDM5D	0.17 (-2.03 - 1.03)	0.19 (-2.03 - 1.03)	-1.66 (-2.03 - 0.83)	0.188
MYST1	0.02 (-2.42 - 4.43)	0.17 (-1.91 - 4.43)	-0.93 (-2.42 - 0.61)	**<0.001**
SETD8	-0.01 (-3.63 - 4.09)	0.14 (-3.63 - 4.09)	-0.25 (-2.15 - 1.40)	**0.026**
SETDB1	-0.13 (-2.71 - 3.53)	-0.13 (-2.71 - 3.53)	-0.13 (-1.88 - 1.70)	0.719
SIRT2	-0.08 (-2.96 - 3.28)	-0.04 (-2.96 - 3.28)	-0.31 (-1.49 - 1.28)	**0.043**
SUV39H1	-0.05 (-2.54 - 3.91)	-0.01 (-2.54 - 3.91)	-0.24 (-1.80 - 0.74)	**0.008**
SUV39H2	-0.06 (-2.47 - 3.62)	-0.22 (-2.47 - 3.62)	0.76 (-0.56 - 1.70)	**<0.001**
SUV420H2	0.02 (-3.37 - 3.32)	0.17 (-1.25 - 3.32)	-1.31 (-3.37 - 0.08)	**<0.001**
TET3	0.23 (-4.94 - 1.55)	0.15 (-4.94 - 1.55)	0.42 (-1.05 - 1.37)	**0.021**

*P*-value is estimated using Wilcoxon rank-sum test and Chi-squared test.

**Table 5:** Baseline characteristics of risk subgroups in gene cluster 1 (n=177).

Characteristics	Gene cluster 1 (GC1)		
	Low-risk, n = 169	High-risk, n = 8	*P*
Diagnosis age	65 (35 - 88)	73 (57 - 82)	0.065
Sex			0.074
Female	79 (46.7%)	1 (12.5%)	
Male	90 (53.3%)	7 (87.5%)	
Ethnicity			0.288
Hispanic Or Latino	5 (3.0%)	0 (0.0%)	
Not Hispanic Or Latino	126 (74.6%)	4 (50.0%)	
Unknown	38 (22.5%)	4 (50.0%)	
Race			0.183
Asian	9 (5.3%)	2 (25.0%)	
Black or African American	6 (3.6%)	0 (0.0%)	
White	150 (88.8%)	6 (75.0%)	
Unknown	4 (2.4%)	0 (0.0%)	
Histology			0.359
8500/3	134 (79.3%)	8 (100.0%)	
Others	35 (20.7%)	0 (0.0%)	
Pathological stage			1.000
Stage 1	20 (11.9%)	1 (14.3%)	
Stage 2	140 (83.3%)	6 (85.7%)	
Stage 3	4 (2.4%)	0 (0.0%)	
Stage 4	4 (2.4%)	0 (0.0%)	
Unknown	1	1	
LN+	119 (70.4%)	4 (50.0%)	0.249
Treatment			0.705
CT (Gemcitabine-based)	68 (40.2%)	2 (25.0%)	
RT+CT (Gemcitabine-based)	22 (13.0%)	2 (25.0%)	
CT (Others)	8 (4.7%)	0 (0.0%)	
RT+CT (Others)	14 (8.3%)	1 (12.5%)	
RT alone	4 (2.4%)	0 (0.0%)	
None	53 (31.4%)	3 (37.5%)	
Progressed disease	97 (57.4%)	6 (75.0%)	0.471
Died	87 (51.5%)	5 (62.5%)	0.722

*P*-value is estimated using Wilcoxon rank-sum test and Fisher's exact test.

**Table 6:** Baseline characteristics of risk subgroups in gene cluster 2 (n=177).

Characteristics	Gene cluster 2 (GC2)		
	Low-risk, n = 144	High-risk, n = 33	*P*
Diagnosis age	65 (35 - 88)	64 (41 - 84)	0.177
Sex			0.232
Female	62 (43.1%)	18 (54.5%)	
Male	82 (56.9%)	15 (45.5%)	
Ethnicity			0.097
Hispanic Or Latino	3 (2.1%)	2 (6.1%)	
Not Hispanic Or Latino	103 (71.5%)	27 (81.8%)	
Unknown	38 (26.4%)	4 (12.1%)	
Race			**0.007**
Asian	10 (6.9%)	1 (3.0%)	
Black or African American	3 (2.1%)	3 (9.1%)	
White	130 (90.3%)	26 (78.8%)	
Unknown	1 (0.7%)	3 (9.1%)	
Histology			0.46
8500/3	114 (79.2%)	28 (84.8%)	
Others	30 (20.8%)	5 (15.2%)	
Pathological stage			0.336
Stage 1	17 (12.0%)	4 (12.1%)	
Stage 2	120 (84.5%)	26 (78.8%)	
Stage 3	3 (2.1%)	1 (3.0%)	
Stage 4	2 (1.4%)	2 (6.1%)	
Unknown	2	0	
LN+	99 (68.8%)	24 (72.7%)	0.654
Treatment			0.482
CT (Gemcitabine-based)	58 (40.3%)	12 (36.4%)	
RT+CT (Gemcitabine-based)	20 (13.9%)	4 (12.1%)	
CT (Others)	8 (5.6%)	0 (0.0%)	
RT+CT (Others)	13 (9.0%)	2 (6.1%)	
RT alone	4 (2.8%)	0 (0.0%)	
None	41 (28.5%)	15 (45.5%)	
Progressed disease	77 (53.5%)	26 (78.8%)	**0.008**
Died	65 (45.1%)	27 (81.8%)	**<0.001**

*P*-value is estimated using Wilcoxon rank-sum test, Chi-squared test, and Fisher's exact test.

**Table 7:** Cox regression analysis results for PFS and OS.

Characteristics	Comparison	Crude-HR (95% CI)	*P*	Adjust-HR^a^ (95% CI)	*P*
		**Survival outcome: PFS**			
Gene cluster 1	High-risk vs low risk	2.05 (0.89, 4.72)	0.092	1.82 (0.72, 4.58)	0.200
Gene cluster 2	High-risk vs low risk	**1.68 (1.07, 2.63)**	**0.025**	**1.69 (1.06, 2.69)**	**0.028**
Age	years	1.01 (0.99, 1.03)	0.180	-	
Sex	Male vs female	0.97 (0.66, 1.43)	0.880	-	
Ethnicity	Non-vs hispanic Or Latino	1.3 (0.41, 4.15)	0.650	-	
	Others vs hispanic Or Latino	1.69 (0.51, 5.58)	0.390	-	
Race	Black/African American vs Asian	1.1 (0.31, 3.91)	0.880	-	
	Others vs Asian	1.45 (0.41, 5.13)	0.570	-	
	White vs Asian	1.07 (0.47, 2.44)	0.880	-	
Histology	Others vs 8500/3	**0.38 (0.21, 0.69)**	**0.001**	0.56 (0.28, 1.08)	0.085
Pathological stage	Stage 4 vs 1	**2.95 (1.35, 6.48)**	**0.007**	1.75 (0.78, 3.93)	0.200
	Stage 4 vs 2	2.36 (0.48, 11.5)	0.290	0.8 (0.10, 6.65)	0.800
	Stage 4 vs 3	2.85 (0.72, 11.2)	0.130	1.5 (0.30, 7.47)	0.600
LN	LN+ vs LN-	**2.04 (1.30, 3.21)**	**0.002**	-	
Treatment	CT+RT vs CT only	0.7 (0.43, 1.14)	0.150	-	
	Others vs CT only	0.81 (0.51, 1.29)	0.380	-	
		**Survival outcome: OS**			
Gene cluster 1	High-risk vs low risk	1.23 (0.50, 3.03)	0.650	-	
Gene cluster 2	High-risk vs low risk	**1.89 (1.20, 2.97)**	**0.006**	1.57 (0.96, 2.58)	0.075
Age	years	**1.03 (1.01, 1.05)**	**0.010**	**1.03 (1.01, 1.05)**	**0.008**
Sex	Male vs female	0.81 (0.54, 1.23)	0.330	-	
Ethnicity	Non-vs hispanic Or Latino	1.52 (0.47, 4.89)	0.480	-	
	Others vs hispanic Or Latino	1.52 (0.45, 5.17)	0.500	-	
Race	Black/African American vs Asian	1.23 (0.33, 4.61)	0.750	-	
	Others vs Asian	1.29 (0.31, 5.41)	0.730	-	
	White vs Asian	1.27 (0.51, 3.14)	0.610	-	
Histology	Others vs 8500/3	**0.41 (0.22, 0.77)**	**0.006**	**0.47 (0.23, 0.93)**	**0.031**
Pathological stage	Stage 4 vs 1	**2.34 (1.07, 5.09)**	**0.033**	-	
	Stage 4 vs 2	1.05 (0.13, 8.60)	0.960	-	
	Stage 4 vs 3	2.15 (0.44, 10.5)	0.350	-	
LN	LN+ vs LN-	**2.19 (1.33, 3.62)**	**0.002**	**2.03 (1.20, 3.43)**	**0.008**
Treatment	CT+RT vs CT only	0.71 (0.40, 1.26)	0.240	0.73 (0.41, 1.30)	0.300
	Others vs CT only	1.52 (0.96, 2.39)	0.074	**2.10 (1.28, 3.45)**	**0.003**

^a^ Adjust-HR is estimated using multivariate Cox regression, the included variables are selected via stepwise a selection procedure.
