# Supplementary material for: Clustering of Chromatin Remodeling Enzymes Predicts Prognosis and Clinical Benefit of Therapeutic Strategy in Pancreatic Cancer
Source: Int J Med Sci. 2022 Sep 21;19(10):1615–27. doi: 10.7150/ijms.73800 (PMC9515693; doi:10.7150/ijms.73800)
Supplement: Supplementary file 1 — Supplementary figures. [file ijmsv19p1615s1.pdf]

Supplementary Figure S1. Boxplot of RNA-seq expression for each of the candidate genes in the study population according to progression-free survival status. *P*-value is estimated using the Wilcoxon rank sum test. DF, disease-free. PD, progressed disease.

Supplementary Figure S2. Boxplot of RNA-seq expression for each of the candidate genes in the study population according to overall survival status. *P*-value is estimated using the Wilcoxon rank sum test. DF, disease-free. PD, progressed disease.

Supplementary Figure S3. Forest plot for risk estimation based on gene expression associated with chromatin remodeling. *Results from the current study. GC: gene cluster, exp.: expression, AML: acute myeloid leukemia, TE: effect size, SE: standard error, HR: hazard ratio, CI: confidence interval, PFS: progression-free survival, DFS: disease-free survival, OS: overall survival.

sFigure 1.

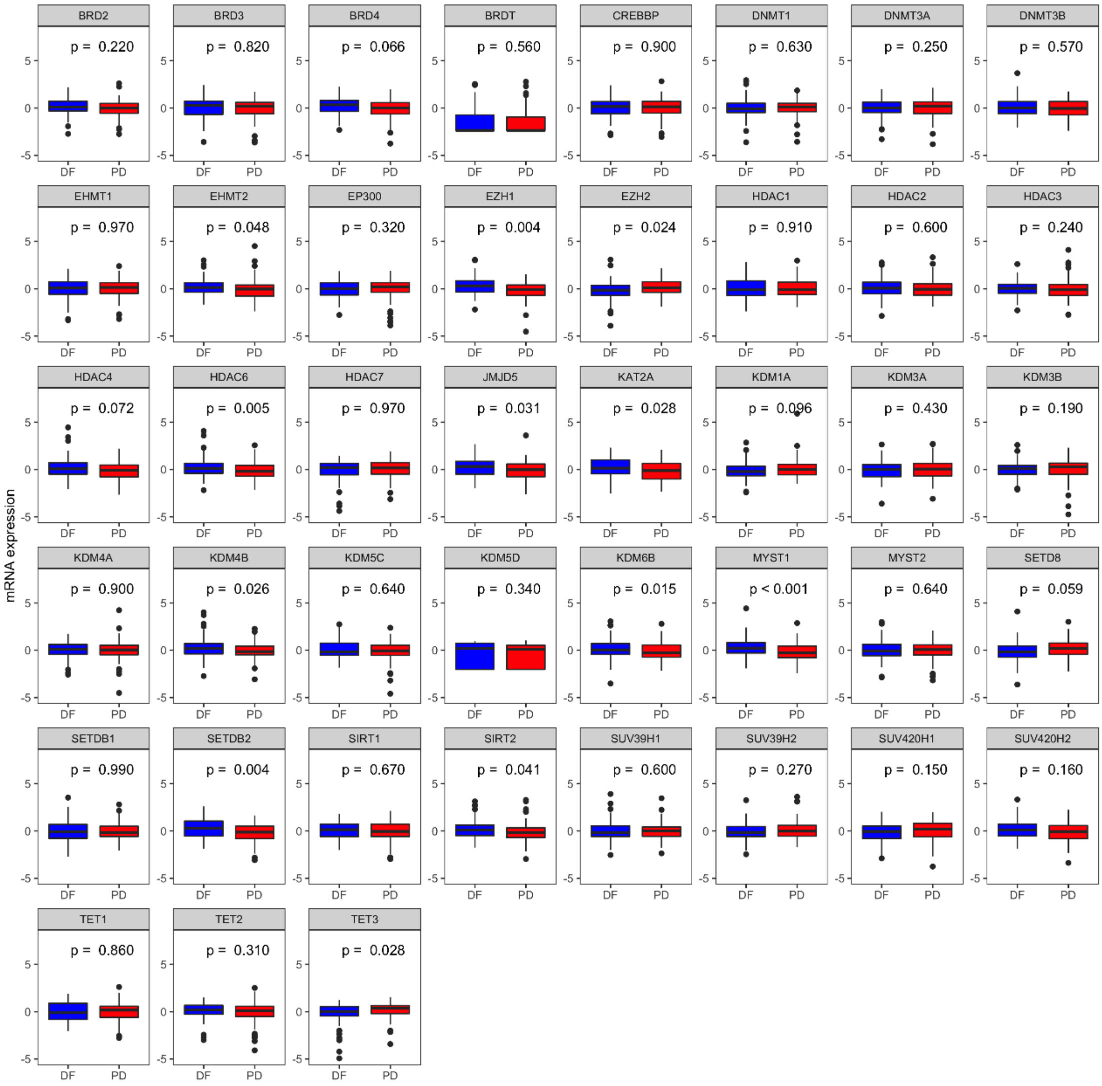
mRNA expression
BRD2 p = 0.220
BRD3 p = 0.820
BRD4 p = 0.066
BRDT p = 0.560
CREBBP p = 0.900
DNMT1 p = 0.630
DNMT3A p = 0.250
DNMT3B p = 0.570
EHMT1 p = 0.970
EHMT2 p = 0.048
EP300 p = 0.320
EZH1 p = 0.004
EZH2 p = 0.024
HDAC1 p = 0.910
HDAC2 p = 0.600
HDAC3 p = 0.240
HDAC4 p = 0.072
HDAC6 p = 0.005
HDAC7 p = 0.970
JMJD5 p = 0.031
KAT2A p = 0.028
KDM1A p = 0.096
KDM3A p = 0.430
KDM3B p = 0.190
KDM4A p = 0.900
KDM4B p = 0.026
KDM5C p = 0.640
KDM5D p = 0.340
KDM6B p = 0.015
MYST1 p < 0.001
MYST2 p = 0.640
SETD8 p = 0.059
SETDB1 p = 0.990
SETDB2 p = 0.004
SIRT1 p = 0.670
SIRT2 p = 0.041
SUV39H1 p = 0.600
SUV39H2 p = 0.270
SUV420H1 p = 0.150
SUV420H2 p = 0.160
TET1 p = 0.860
TET2 p = 0.310
TET3 p = 0.028
DF
PD
5
0
-5

sFigure 2.

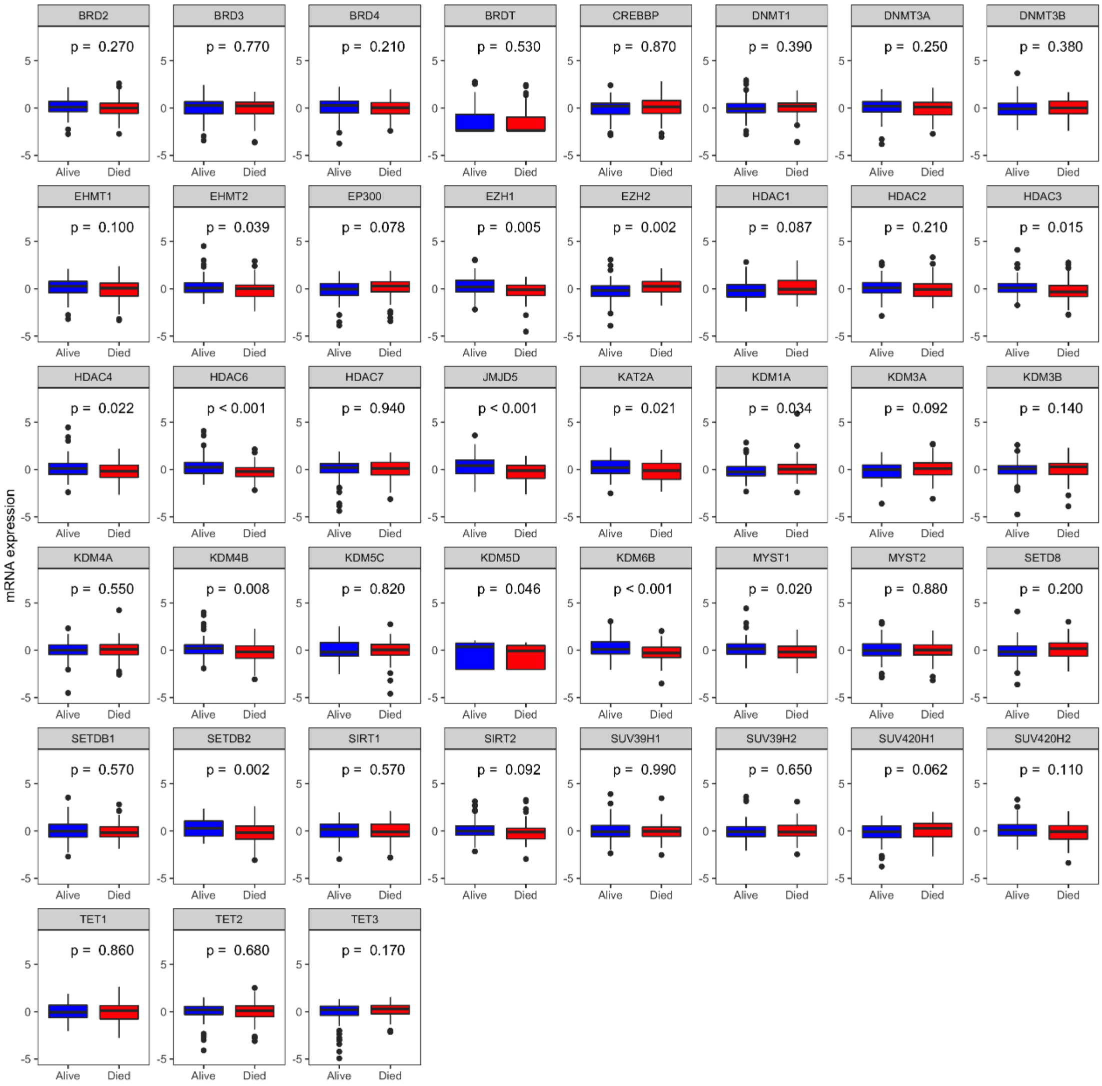

sFigure 3.

Study	TE	seTE	HR	95%-CI	Weight (common)	Weight (random)
**Outcome = Disease progression**						
*Wang: 2022: Pancreatic (PFS): mRNA: GC1: high-vs. low-risk	0.60	0.4720	1.82	[0.72; 4.59]	3.6%	6.8%
*Wang: 2022: Pancreatic (PFS): mRNA: GC2: high-vs. low-risk	0.52	0.2376	1.69	[1.06; 2.69]	14.4%	10.9%
R.Wieczorek: 2015: Colorectal (DFS): mRNA: TET1: intermediate-vs. low-exp.	1.69	0.8004	5.40	[1.12; 25.93]	1.3%	3.5%
R.Wieczorek: 2015: Colorectal (DFS): mRNA: TET1: high-vs. low-exp.	0.71	0.9397	2.03	[0.32; 12.80]	0.9%	2.7%
R.Wieczorek: 2015: Colorectal (DFS): mRNA: TET2: intermediate-vs. low-exp.	-0.67	0.5305	0.51	[0.18; 1.44]	2.9%	6.0%
R.Wieczorek: 2015: Colorectal (DFS): mRNA: TET2: high-vs. low-exp.	-1.56	0.8360	0.21	[0.04; 1.08]	1.2%	3.3%
Zhang: 2020: AML (DFS): miRNA: TET3: low- vs. high-exp.	-0.46	0.2057	0.63	[0.42; 0.95]	19.2%	11.5%
Yang: 2015: Breast (DFS): mRNA: TET1: low- vs. high-exp.	0.50	0.3219	1.65	[0.88; 3.11]	7.8%	9.3%
Yang: 2015: Breast (DFS): mRNA: TET2: low- vs. high-exp.	0.60	0.3232	1.82	[0.96; 3.42]	7.8%	9.3%
Yang: 2015: Breast (DFS): mRNA: TET3: low- vs. high-exp.	0.66	0.3245	1.94	[1.03; 3.67]	7.7%	9.3%
**Common effect model**			1.23	[0.99; 1.52]	66.7%	--
**Random effects model**			1.31	[0.87; 1.98]	--	72.5%
Heterogeneity: $I^2 = 68\%$, $\tau^2 = 0.2461$, $p < 0.01$						
**Outcome = Overall survival**						
*Wang: 2022: Pancreatic (OS): mRNA: GC2: high-vs. low-risk	0.45	0.2522	1.57	[0.96; 2.57]	12.8%	10.6%
Zhang: 2020: AML (OS): miRNA: TET3: low- vs. high-exp.	-0.44	0.2118	0.64	[0.43; 0.98]	18.1%	11.4%
Yang: 2015: Breast (OS): mRNA: TET3: low- vs. high-exp.	0.64	0.5746	1.90	[0.62; 5.87]	2.5%	5.5%
**Common effect model**			0.98	[0.72; 1.33]	33.3%	--
**Random effects model**			1.14	[0.57; 2.28]	--	27.5%
Heterogeneity: $I^2 = 77\%$, $\tau^2 = 0.2626$, $p = 0.01$						
**Common effect model**			**1.14**	**[0.95; 1.36]**	**100.0%**	**--**
**Random effects model**			**1.26**	**[0.89; 1.77]**	**--**	**100.0%**

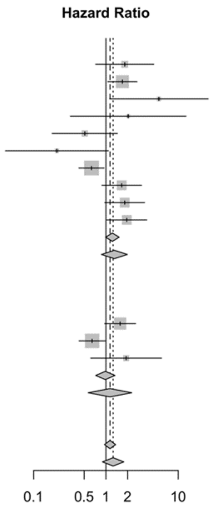

Heterogeneity: $I^2 = 69\%$, $\tau^2 = 0.2191$, $p < 0.01$
Test for subgroup differences (common effect): $\chi^2_1 = 1.36$, df = 1 ($p = 0.24$)
Test for subgroup differences (random effects): $\chi^2_1 = 0.12$, df = 1 ($p = 0.73$)
